# Closing the system: production of viral antigen-presenting dendritic cells eliciting specific CD8^+^ T cell activation in fluorinated ethylene propylene cell culture bags

**DOI:** 10.1186/s12967-020-02543-1

**Published:** 2020-10-09

**Authors:** Jean-Philippe Bastien, Natalie Fekete, Ariane V. Beland, Marie-Paule Lachambre, Veronique Laforte, David Juncker, Vibhuti Dave, Denis-Claude Roy, Corinne A. Hoesli

**Affiliations:** 1 Department of Chemical Engineering, McGill University, Montreal, Québec Canada; 2 Hematology-Oncology and Cell Therapy Institute, Hopital Maisonneuve-Rosemont Research Center, Montreal, Québec Canada; 3 Department of Biomedical Engineering, McGill University, Montreal, Québec Canada; 4 McGill Genome Centre, McGill University, Montreal, Québec Canada; 5 Department of Neurology and Neurosurgery, McGill University, Montreal, Québec Canada; 6 Department of Microbiology, Infectiology and Immunology, Université de Montréal, Montreal, Québec Canada; 7 Department of Medicine, Université de Montréal, Montreal, Québec Canada

**Keywords:** Cellular therapy, Dendritic cell, Fluorinated polymers, Immunotherapy, Monocyte, Polystyrene, Scale-down

## Abstract

**Background:**

A major obstacle to anti-viral and -tumor cell vaccination and T cell immunotherapy is the ability to produce dendritic cells (DCs) in a suitable clinical setting. It is imperative to develop closed cell culture systems to accelerate the translation of promising DC-based cell therapy products to the clinic. The objective of this study was to investigate whether viral antigen-loaded monocyte-derived DCs (Mo-DCs) capable of eliciting specific T cell activation can be manufactured in fluorinated ethylene propylene (FEP) bags.

**Methods:**

Mo-DCs were generated through a protocol applying cytokine cocktails combined with lipopolysaccharide or with a CMV viral peptide antigen in conventional tissue culture polystyrene (TCPS) or FEP culture vessels. Research-scale (< 10 mL) FEP bags were implemented to increase R&D throughput. DC surface marker profiles, cytokine production, and ability to activate antigen-specific cytotoxic T cells were characterized.

**Results:**

Monocyte differentiation into Mo-DCs led to the loss of CD14 expression with concomitant upregulation of CD80, CD83 and CD86. Significantly increased levels of IL-10 and IL-12 were observed after maturation on day 9. Antigen-pulsed Mo-DCs activated antigen-responsive CD8^+^ cytotoxic T cells. No significant differences in surface marker expression or tetramer-specific T cell activating potency of Mo-DCs were observed between TCPS and FEP culture vessels.

**Conclusions:**

Our findings demonstrate that viral antigen-loaded Mo-DCs produced in downscaled FEP bags can elicit specific T cell responses. In view of the dire clinical need for closed system DC manufacturing, FEP bags represent an attractive option to accelerate the translation of promising emerging DC-based immunotherapies.

## Background

Cell culture in disposable, functionally closed bioreactor bag systems is of major interest for cell-based therapy because it minimizes the risks of contamination, limits cell exposure to shear stress, and can be scaled up to obtain therapeutic doses of cells [1]. Safe, economical and reliable cell culture systems that are in full compliance with the recommendations of regulatory authorities are essential. The implementation of closed culture systems can reduce space and clean room requirements for cell production under current good manufacturing practice, thereby reducing the costs required for clinical grade therapeutic cell production. Moreover, culture bags are needed in order to incorporate automation.

One cell therapy product of major commercial and clinical interest is dendritic cells (DCs) [2]. Indeed, DCs have been hypothesized to be the panacea for personalized immunotherapy owing to their unique capacity to activate T cells and initiate the adaptive immune response to specifically eliminate target cells that are transformed or infected with pathogens [2–4]. Due to their low frequency of occurrence in peripheral blood, DCs are commonly differentiated ex vivo from monocytes in the presence of cytokines added to culture media, typically interleukin (IL)-4 and macrophage-colony stimulating factor (GM-CSF) [5]. The immature monocyte-derived DCs (Mo-DCs) thus generated are induced to mature when loaded with peptide antigen, usually in combination with toll-like receptor-activating inflammatory cytokine mixtures of varying composition [6].

For Mo-DC generation, cell culture bags are ideal as they can easily accommodate clinical-scale volumes of starting and in-process material which are typically of 100 mL or more [7–12]. Fluorinated ethylene propylene (FEP) bags are of particular interest as they are optically clear, allow gas exchange in cell culture incubators, are chemically inert, facilitate cell recovery, and remain flexible at temperatures ranging from − 240 to + 205 °C, making them outstanding candidates for cell processing, cell culture and cryopreservation. Nevertheless, most translational research studies are performed in tissue culture polystyrene (TCPS)-based plates or flasks of much smaller volumes (typically ≤ 10 mL) and conducted in rigid tissue culture polystyrene (TCPS)-based plates or flasks. As recently reviewed by Fekete et al*.* [13], the transition from functionally-open TCPS plates to closed systems such as FEP or polyolefin bags leads to a concurrent transition in material properties including gas permeability, mechanical properties, surface topography, surface chemistry and surface wettability. This may affect protein adsorption profiles and resulting changes in the cell microenvironment which may impact Mo-DC cell fate decisions, as observed with other therapeutic cells [14].

A number of groups have reported successful production of Mo-DCs in FEP bags based on the upregulation of DC markers and on the capacity to stimulate T cells [15–19]. The number of direct comparative studies between TCPS plates and FEP bags is however much more limited [9, 11, 12]. Most studies comparing TCPS flasks with FEP or other types of hydrophobic culture bags report no marked changes in Mo-DC differentiation [7–9, 11, 12, 20, 21]. However, subtle differences in cytokine production [9] and the expression levels of certain surface markers such as CD1a [7, 22] have been reported. The impact of these differences on antigen-specific T cell activation, a key function of Mo-DC, and hence product potency has not been thoroughly assessed [22]. The lack of commercially available research-scale culture bags limited the throughput of past comparative studies, and hence the dynamics of the DC differentiation process in FEP bags have not been reported. Together, these limitations result in a gap in our understanding of cell-material interactions early in the upscaling process and thus, in bag usage in the clinical setting.

The main objective of this study was to compare the phenotype and functional capacities of Mo-DCs cultured in ‘open’ TCPS-based plates to the ‘closed’ fluorinated ethylene propylene (FEP) culture bag systems. Research-scale FEP bags were tested, providing a novel platform for translational studies using cell culture materials more similar to clinical-scale cultures. Mo-DCs generated in FEP bags and TCPS plates showed comparable levels of antigenic expression and cytokine production and were able to efficiently induce tetramer-specific effector T cell response upon viral antigen stimulation.

## Methods

### Culture surfaces

Immature as well as mature Mo-DCs were cultured in Nunclon™ Delta-treated TCPS 24-multiwell plates (Nunc, ThermoFisher) or untreated VueLife^®^ FEP culture bags (Saint-Gobain) of 1 mL (1PF-0001), 2 mL (2PF-0002) and 7 mL (1PF-0007) volumes. The respective internal dimensions of the bags were approximately 3.8 cm × 2 cm, 2.5 cm × 8.6 cm or 3.4 cm × 5.8 cm with a single Luer-lock cell seeding and medium exchange port. These bags are commercialized for cell cryopreservation applications but can also be used for cell culture.

### Generation of Mo-DCs using lipopolysaccharides to induce maturation

**Fig. 1:**
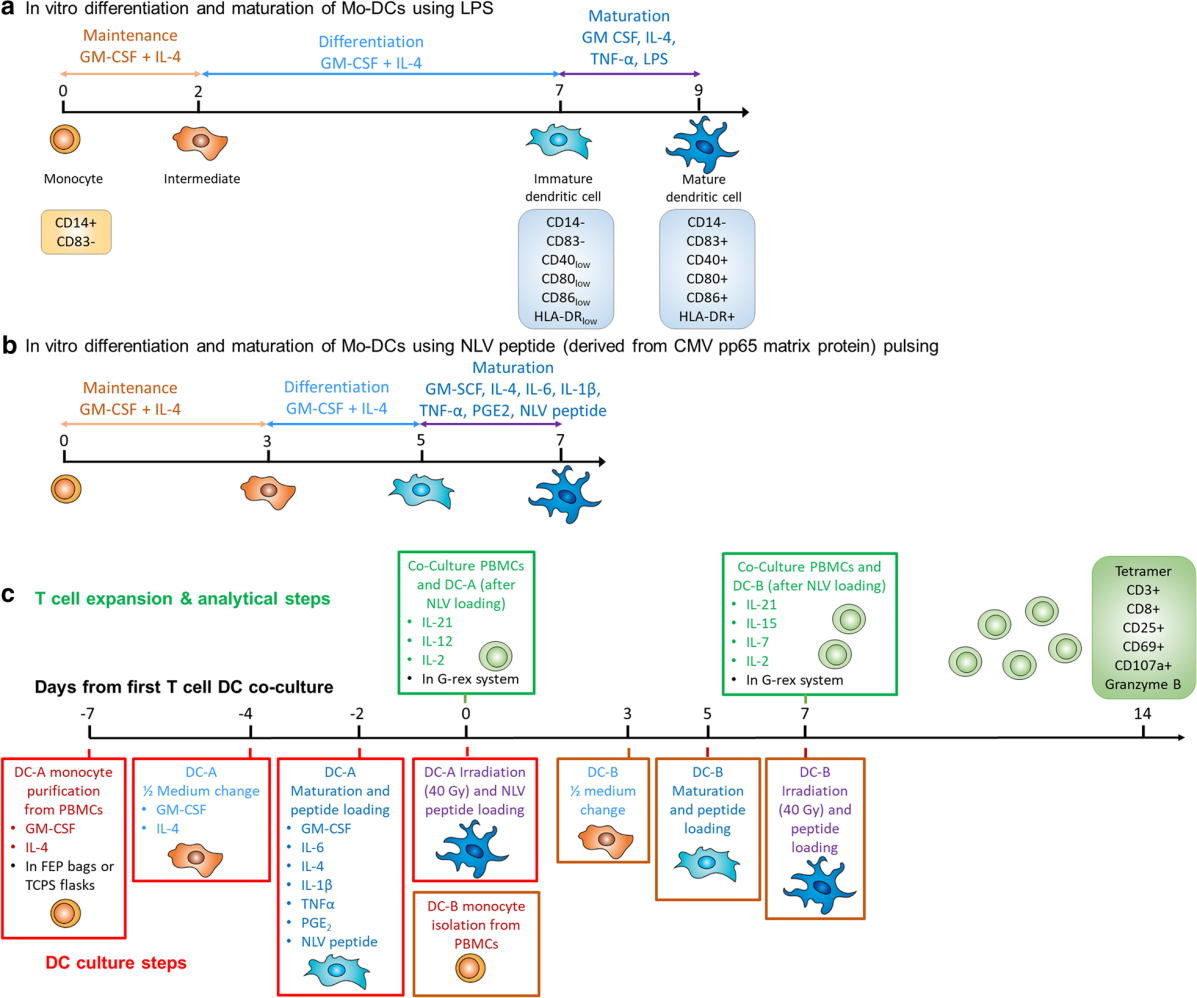
Overview of cell culture methods for Mo-DC production, phenotypic and functional assessment. **a** Mo-DC differentiation protocol with maturation in the presence of LPS. **b** Mo-DC differentiation protocol with NLV peptide (derived from CMV pp65 matrix protein) loading. **c** T cell and Mo-DC co-culture protocol to determine the ability to generate specific T cell using NLV-loaded DCs produced in two batches (DC-A and DC-B) for T cell re-stimulation

CD14-positive monocytes were freshly isolated from peripheral whole blood of healthy human donors. Peripheral blood was subjected to gradient density centrifugation using Histopaque^®^-1077 (Sigma Aldrich) in SepMate™-50 tubes (STEMCELL). CD14-positive cells were isolated via magnetic cell sorting using MACS® Technology with CD14 Microbeads, LS columns and a MidiMACS™ Separator (Miltenyi Biotec, Bergisch Gladbach, Germany) according to manufacturer’s instructions. CD14-positive cells were then seeded at a density of 1.5 × 10^6^ cells/ml (~ 0.5 mL/cm^2^ bottom surface area) onto TCPS or FEP culture surfaces in GMP DC medium (CellGenix GmbH, Freiburg, Germany) supplemented with 1000 IU/ml of GM-CSF and 1000 IU/ml of IL-4 (both from Miltenyi Biotec) and cultured at 37 °C, 5% CO_2_ for 7 days to obtain immature DCs. Adherent and non-adherent cells from TCPS plates were harvested via incubation with TrypLE Express (Thermo Fisher Scientific) for 5 min at 37 °C, 5% CO_2_, followed by centrifugation at 300×*g* for 5 min. FEP bags display a non-adherent surface and did not require trypsin exposure to recover cells. To induce maturation, Mo-DCs were cultured 2 additional days in CellGenix^®^ GMP DC medium supplemented with 1000 U/mL GM-CSF, 100 U/mL IL-4 and 100 μg/ml of Toll-like receptor-validated lipopolysaccharides (LPS) (Sigma Aldrich) and 10 ng/mL of purified human TNF-α (R&D Systems). The cells obtained using this maturation cocktail are referred to as “LPS treated Mo-DCs” (Fig. 1a). Microphotographs of cells during culture were taken using an inverted phase contrast microscope (Trinocular Inverted Microscope, VWR).

### Generation of antigen-pulsed Mo-DCs

After PBMC collection and CD14^+^ cell enrichment as described above, CD14^+^ cells were seeded in FEP bags or TCPS plates at a concentration of 2 × 10^6^ cells/mL in RPMI medium (Gibco) supplemented with 10% FBS, 1 mM sodium pyruvate, non-essential amino acids, 2 μM l-glutamine, 100 U/mL penicillin, 100 μg/mL streptomycin (all Gibco, Burlington, ON, Canada), 50 µM β-mercaptoethanol (Sigma-Aldrich) in the presence of 800 IU/mL GM-CSF and 1000 IU/mL IL-4 (both from Miltenyi Biotec) and cultured at 37 °C, 5% CO_2_ for 5 days with medium replenishment at day 3 to obtain immature DCs. In multiwell plates, adherent and non-adherent cells were harvested by gently scraping adherent cells followed by centrifugation at 300×*g* for 10 min. For culture bags, adherent and non-adherent cells were harvested by massaging the bags and manually moving culture medium up and down using a syringe connected to the bag port. Next, DC maturation was induced by 2 days of culture in the presence of 800 U/mL GM-CSF, 100 ng/mL IL-6, 1000 U/mL IL-4, 10 ng/mL IL-1β, 10 ng/mL TNF-α, 1 µg/mL PGE_2_ (all Miltenyi Biotec) and 1 µg/mL of HLA-A02 restricted epitope derived from CMV pp65 matrix protein NLVPMVATV (NLV) peptide (Genescript). For TCPS cultures, adhering cells were harvested using cell scraping. Cells were then pulsed for 4 h in the presence of 1 µg/mL NLV peptide. We refer to these cells as “pulsed Mo-DCs” (Fig. 1b).

### T cell activation using pulsed Mo-DCs

T cells were obtained from the negative fraction following CD14 selection. Next, 15 × 10^6^ T cells were co-cultured in the presence of unpulsed or antigen-pulsed irradiated Mo-DCs at a 10:1 ratio (T cell:Mo-DC) in G-Rex^®^10 bioreactors (Wilson Wolf) for a total of 14 days (Fig. 1c). T cells cultured in the presence of antigenic peptide alone (1 µg/mL) served as control. For the first 7 days of co-culture, T cells were cultured in the presence of 30 ng/mL IL-21, 10 ng/mL IL-12, and 100 U/mL IL-2 (all from Miltenyi Biotech) added to RPMI medium (Gibco) supplemented with 10% FBSI, 1 mM sodium pyruvate, 1X MEM nonessential amino acids, 2 μM l-glutamine, 100 U/mL penicillin, 100 μg/mL streptomycin (all Gibco, Burlington, ON, Canada), and 50 µM β-mercaptoethanol (Sigma-Aldrich). At day 7, T cells underwent a second round of stimulation with pulsed (or unpulsed) Mo-DCs. T cells were collected and suspended in fresh culture medium containing IL-21 (30 ng/mL), IL-15 (5 ng/mL), IL-7 (10 ng/mL) and IL-2 (100 U/mL) (all from Miltenyi Biotech). Activation and function of T cells was analysed on day 3, 7, 10 and 14 of culture.

### Cell enumeration

Adherent cells, harvested using TrypLE™ Express (Life Technologies), as well as suspension cells were enumerated using a 0.1 mm depth hemocytometer (Bright-Line, Hausser Scientific) or via particle counts using a Bio-rad TC20 automated cell counter (Bio-rad).

### Flow cytometry

Flow cytometry was performed using a FACSCalibur™ (Becton Dickinson) equipped with CellQuest™ Software v3.3 for Mo-DC differentiation assays and a BD Fortessa x-20 (BD Biosciences) equipped with FACSDiva™ Software v8.0 for all T cell phenotyping studies. Antibodies used for Mo-DC characterization included CD1a (HI149), CD14 (MφP9), CD40 (5C3), CD80 (L307.4), CD83 (HB15e), CD86 (2331), CD197 (150503), HLA-DR (G46-6; all from BD Biosciences) and CD54 (HA58; BioLegend). The BD Horizon™ Fixable Viability Stain 660 (BD Biosciences) was used for live/dead discrimination. Antibodies used for T cell characterization included CD3 (SK7), CD4 (SK3), CD8 (SK1), CD25 (BC96), CD107a (H4A3), Granzyme B (GB11), HLA-DR (G46-6) (all BD Biosciences), and CD45RA (HI100) (all from Biolegend, San Diego, CA, USA). Cells were collected by combining suspended and adherent cells in a conical tube and re-suspending in FACS buffer consisting of 0.22 µm filtered phosphate-buffered saline solution (without Ca^2+^ and Mg^2+^, Life Technologies) containing 0.5% bovine serum albumin (heat shock fraction, Sigma-Aldrich) and 2 mM ethylenediaminetetraacetic acid (Life Technologies). For studies with peptide-pulsed Mo-DCs, 2 × 10^6^ cultured/activated T cells were either left unstimulated or restimulated for 4 h using CMVpp65 antigen (NLV) in the presence of CD107a antibody. For Granzyme B expression analysis, cells were incubated in the presence of 1X Brefeldin A (BFA) (eBioscience) for the last 2 h of restimulation. For tetramer staining, 2 × 10^6^ cells were harvested, first exposed to 5 nM dasatinib (Sigma) for 30 min at 37 °C, and then exposed to NLV specific tetramer (NIH tetramer core facility, Bethesda, MA) for 40 min at 4 °C. Cells were then washed, incubated with Fc blocking solution (BD Biosciences) and further stained for surface markers. For intracellular staining, T cells were first stained for surface markers, washed and re-suspended in fixation and permeabilization buffer (BD Biosciences) according to the manufacturer instructions and stained for intracellular markers. All reagents were used according to manufacturers’ instructions. Acquired flow cytometry data were further analyzed using FlowJo v10.2 software (FlowJo, LLC).

### Cytokine production

Cell culture supernatants and media samples were collected at day 2, 7 and 9 of culture and stored at − 80 °C until analysis. Levels of human IL-10 and IL-12p40 in Mo-DC culture supernatants were determined using enzyme-linked immunosorbent assay (ELISA) SimpleStep kits (Abcam. IL-6, platelet-derived growth factor (PDGF)-BB, transforming growth factor alpha (TGF-α), matrix metalloproteinase (MMP)-1 and -9 were measured using the antibody colocalization microarray technique described by Laforte et al*.* [23], but without the trehalose spot protection step. In short, capture antibodies from matched pairs used in ELISA assays were immobilized onto activated glass slides. Small volumes of diluted samples were incubated with capture antibodies, followed by deposition of the matched detection antibodies directly atop the capture antibodies. Because each antibody pair is independent, this method avoids most sources of cross-reactivity that leads to false positive signals and allows several proteins to be measured in individual samples.

### Statistics

All statistical analyses were performed using JMP^®^ 11 software (SAS Institute, Cary, NC). ANOVA analysis, post-hoc power analysis and Tukey's HSD post-hoc tests were used to compare the means of cell surface marker expression, viability and cytokine production data. Unless otherwise mentioned, all between-group comparisons were performed using the donor as a blocking variable (i.e. one-way ANOVA). When the number of replicates performed with FEP bags differed from TCPS controls, between-group comparisons were performed using both paired and unpaired ANOVA and post-hoc tests. All p-values shown represent paired comparisons. Results were considered to be statistically significant at p < 0.05 and highly significant at p < 0.001.

## Results

### Impact of culture volume and time on Mo-DC viability in FEP bags or TCPS plates

**Fig. 2:**
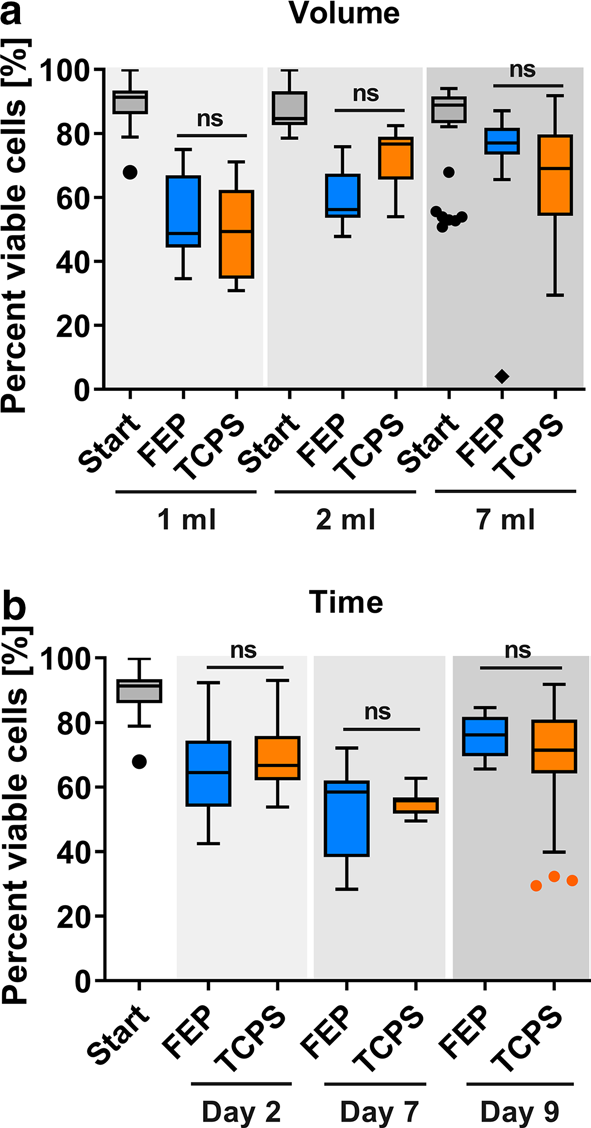
Mo-DC viability in FEP culture bags and TCPS plates. **a** LPS treated Mo-DCs were generated in FEP culture bags (of 1 mL, 2 mL or 7 mL volumes) or 24-multiwell TCPS plates for 9 days (except 2 mL data available only for day 2). **b** Cell viability was assessed before (Start) and after 2, 7 and 9 days of culture in 7 mL FEP bags or TCPS plates. Shown are Tukey's Box-and-whisker plots of 4 to 17 independent experiments per condition. ns: no statistically significant differences for both paired (n ≥ 4) and unpaired comparisons. Using unpaired comparisons, a significantly reduced viability was observed in the 1 mL bags compared to the 2 mL bags (p = 0.02, Tukey HSD test)

The aim of this study was to compare and understand the impact of using TCPS vs FEP as culture material and surface for generating mature Mo-DCs and to assess their capacity to induce antigen-specific T cells. Experiments included different size FEP bags (to assess the effect of bag size/handling) and TCPS flasks (to assess the materials/system effect). We utilized FEP bags of 1 mL, 2 mL and 7 mL volumes and compared the cultures to those performed in TCPS multiwell plates. As shown in Fig. 2a, the mean (± SEM) cell viability after 9 days of culture was overall higher in FEP bags of 2 mL (60 ± 8%) and 7 mL (75 ± 11%) volumes when compared to 1 mL (53 ± 12%) volumes. This effect is likely due to the increased exposure of cells to mechanic stresses while handling the bags for media transfer or cell harvesting during culture in the 1 mL volumes. Based on qualitative observations, the ease of cell handling was significantly improved in bags of 2 mL and larger volumes compared to 1 mL bags where liquid infusion was very challenging due to the tension in the top and bottom films established by the surrounding seals. Cell viability in TCPS plates was comparable to that in 1 mL, 2 mL or 7 mL FEP bags at an overall mean ± SEM of 62 ± 3%. Further, no statistically significant effect could be found when comparing cell viability during culture on either FEP or TCPS surfaces at day 2, 7 or 9 of culture (all FEP bag volumes tested) and TCPS 24-well plates (Fig. 2b). Furthermore, no significant differences in Mo-DC concentration or yield were observed at day 9 with the NLV differentiation protocol between the 7 mL FEP bags and TCPS plates (Additional file 1: Figure S1). A trend towards reduced cell viability was observed from day 2 to day 7 of culture, as is expected for the Mo-DC differentiation process. The subsequent increase in viability (final viability of 73 ± 1% on FEP and 68 ± 2% on TCPS surfaces) could be due to removal of dead cells during the medium exchanges through cell centrifugation at days 3 and 7. In order to standardize the procedure and results, only 7 mL FEP flasks were used in subsequent experiments.

### Surface marker expression of Mo-DCs cultured in FEP bags or TCPS plates

**Fig. 3:**
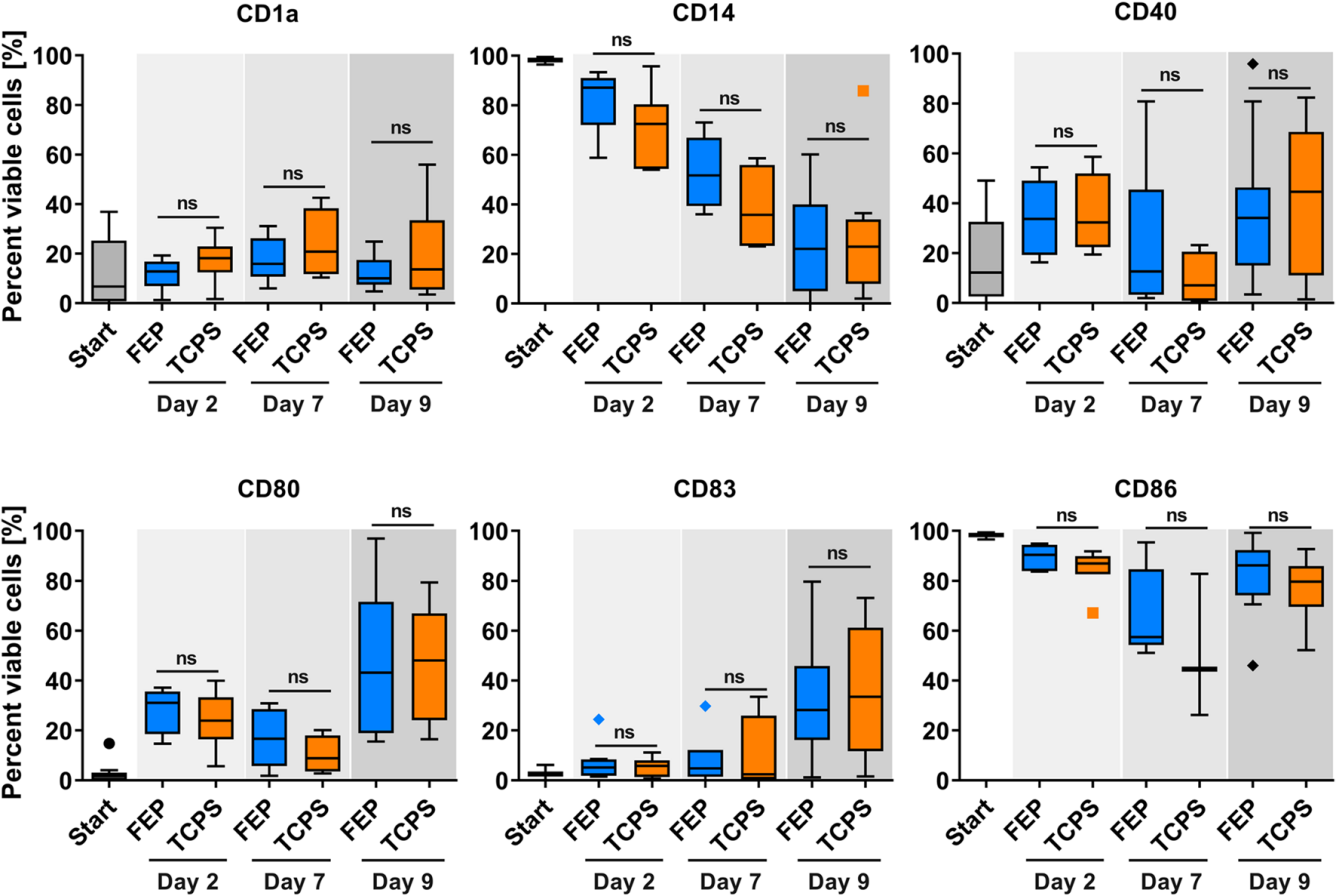
LPS treated Mo-DCs can be efficiently generated in FEP bags and TCPS plates. Flow cytometry was performed before culture (Start) and after 2, 7 and 9 days of differentiation and maturation culture on FEP or TCPS surfaces. Shown are Tukey's Box-and-whisker plots. ns: no statistically significant differences for both paired (n ≥ 4 donors for all surface markers at all time points except n = 3 for CD86 at day 7) and unpaired (n = 14 donors at day 0; n = 4 to 10 donors at other time points except n = 3 for CD86 day 7) comparisons

As cell viability overall was similar when comparing Mo-DCs generated in FEP and TCPS cultures, we characterized the cell phenotype further at different stages of Mo-DC culture. To this end, Mo-DCs were generated in FEP bags (7 mL) or TCPS multiwell plates and analyzed before seeding (day 0), during culture (day 2 and 7) and after maturation (day 9, Fig. 1a) via flow cytometry. As shown in Fig. 3, monocytes could efficiently and comparably be induced to differentiate into immature DCs after 7 days of culture on either FEP or TCPS surfaces. Interestingly, a fraction of CD14-enriched cells adhered to both culture surfaces with progressive detachment as differentiation progressed. After the final maturation step, the adherent cells extended dendrites as would be expected for Mo-DCs (Additional file 1: Figure S2). When comparing the surface marker expression on day 2 vs. day 7, we observed downregulation of CD14 expression, while CD1a and CD83 expression increased indicating differentiation of monocytes into immature DCs. DC hallmark surface marker CD83 and co-stimulatory molecules CD40, CD80 and CD86 were upregulated when compared to day 7 of culture, confirming DC maturation upon addition of TNF-α and LPS. The expression of CD54 (ICAM-1) and HLA-DR remained stable (Additional file 1: Figure S3) throughout culture, suggesting that the cultured cells have the capacity of forming immunological synapses both at the monocyte and more mature DC states. The expression levels of Chemokine Receptor 7 (CCR7, CD197) generally increased during culture, but showed high donor-dependent variability during the maturation phase (Additional file 1: Figure S3). Overall, no statistically significant effect of the culture surface, TCSP or FEP, on Mo-DC surface marker expression could be found at any day of the culture.

### Cytokine production by Mo-DCs cultured in FEP bags and TCPS plates

**Fig. 4:**
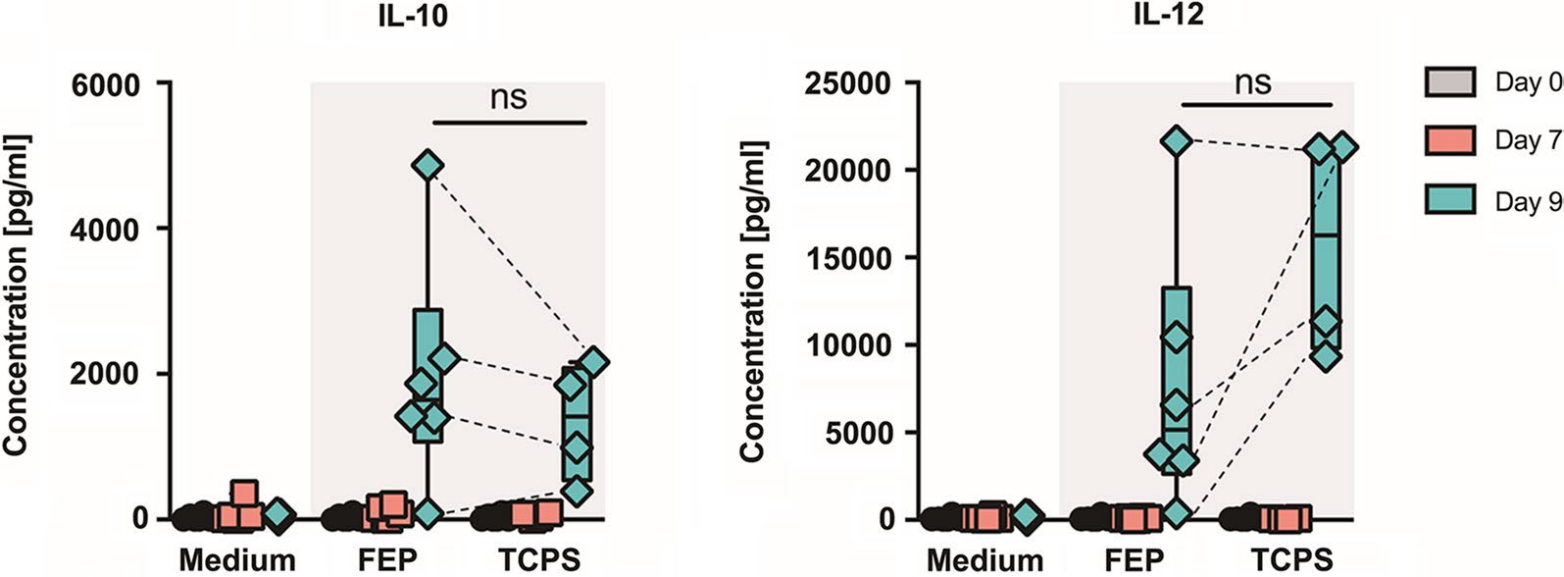
Mo-DC cytokine production in vitro. LPS treated Mo-DCs were generated in FEP culture bags or TCPS plates. Levels of IL-10 and IL-12 in cell culture supernatants collected at day 7 and 9 of culture were compared to medium controls. Shown are min–max Box-and-whisker plots with individual data points from n = 6 donors for FEP and n = 4 donors for TCPS. Dashed lines between day 9 data points represent matched measurements from a given donor. ns: no statistically significant differences for both paired and unpaired comparisons

Mo-DCs are known to produce pro- or anti-inflammatory cytokines to alter the micromilieu and direct immune responses. To determine whether the FEP bag culture system would elicit unexpected spontaneous cytokine release, we measured the production of inflammatory (IL-6, IL-12) and anti-inflammatory (IL-10) cytokines in the culture media of Mo-DCs at days 2 and 7 of DC differentiation. Medium was also collected after maturation and stimulation of the Mo-DCs in the presence of LPS, GM-CSF and TNF-α at day 9. Media samples not exposed to cells were used as controls. As shown in Fig. 4, Mo-DCs started producing IL-10 and IL-12 only upon maturation at day 9 of culture. Similarly, IL-6, platelet-derived growth factor (PDGF)-BB, TGF-α, MMP-1 and MMP-9 were increased in cell culture supernatants at day 7 and 9 of culture in the absence of external stimuli (Additional file 1: Figure S4). No statistically significant differences were observed between cytokine levels detected in Mo-DC cultures on FEP or TCPS surfaces.

### Functional impact of Mo-DC culture conditions on antigen-specific T cell activation potency

**Fig. 5:**
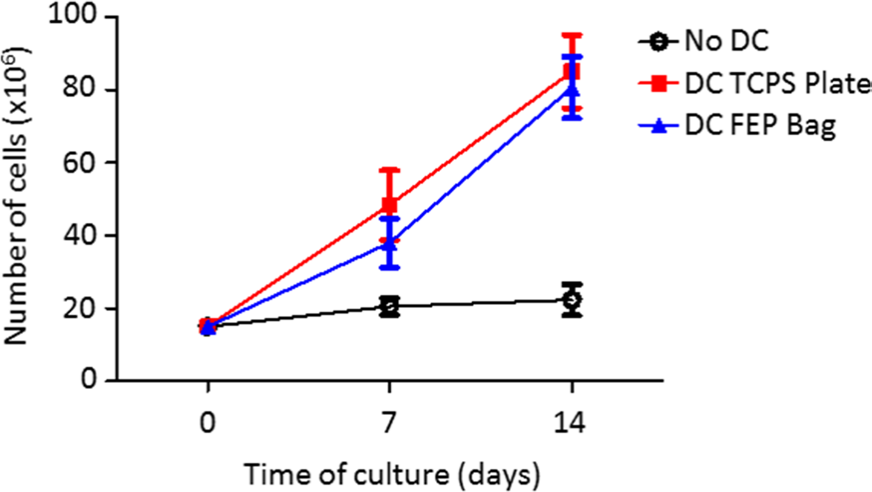
Impact of pulsed Mo-DCs cultured in FEP bags or TCPS plates on T cell expansion. Following Mo-DC differentiation, 15 × 10^6^ T cells were cultured in the presence of CMVpp65 peptide (NLV)-pulsed Mo-DC at 1:10 T cell: Mo-DC ratio. T cells were enumerated at day 0, 7 and 14. Each data point represents the mean ± SEM of 7 independent experiments, each from an independent donor

We next sought to determine whether Mo-DCs generated in FEP bags displayed similar T cell stimulatory capacity compared to Mo-DCs obtained in TCPS plates. In order to perform this evaluation in a clinically-relevant setting, DCs were matured using a combination of TNF-α with IL-6, IL-1β and PGE2, rather than LPS and TNF-α. The combination of these four cytokines has also been shown to produce mature DCs that are more potent than LPS at inducing an inflammatory T cell response [24]. To evaluate if TCPS plates or FEP bags affected Mo-DCs’ capacity to activate and expand antigen-specific T cells, we cultured autologous purified T cells with Mo-DCs differentiated in TCPS plates or FEP bags and pulsed or not with antigen. We observed similar expansion of T cells when cultured in the presence of antigen-pulsed Mo-DCs whether differentiated in TCPS plates or FEP bags (Fig. 5). Next, we evaluated the capacity of pulsed Mo-DCs cultured in FEP bags or in TCPS plates to activate specifically antigen responsive CD8^+^ cytotoxic T cells. For this purpose, we evaluated the activation and frequency of CMV—specific T cells following activation in the presence of CMV pp65 peptide (NLV peptide) pulsed Mo-DCs. After one round (7 day of co-culture) of T cell culture with Mo-DCs, we observed a trend toward an increase in the frequency of tetramer-specific T cells, suggesting expansion of antigen specific CD8^+^ T cells as early as day 7 of co-culture (Additional file 1: Figure S5A, C). We further assessed the activation status of these antigen specific CD8^+^ T cells through their expression of early or later activation marker CD69 and CD25, respectively. Notably, all the tetramer specific T cells detected following co-culture in the presence of peptide-pulsed Mo-DCs expressed the activation marker CD25 cells. In contrast, unpulsed Mo-DCs or NLV peptide alone failed to induce such activation of these cells (Additional file 1: Figure S5B, D). Further, the activation of tetramer-specific CD8^+^ T cells was comparable whether cultured in the presence of peptide-pulsed Mo-DCs differentiated in TCPS plates or in FEP bags. In contrast, only a modest increase of CD25 expression was observed in nonspecific CD4^+^ and CD8^+^Tetramer^−^ T cells cultured in the presence of peptide-pulsed Mo-DC independently of culture vessel (Additional file 1: Figure S6A–F). These results suggest that the MO-DCs cultured in FEP bags are equally capable of antigen-specific T cell activation as MO-DC obtained in TPSC plates.

**Fig. 6:**
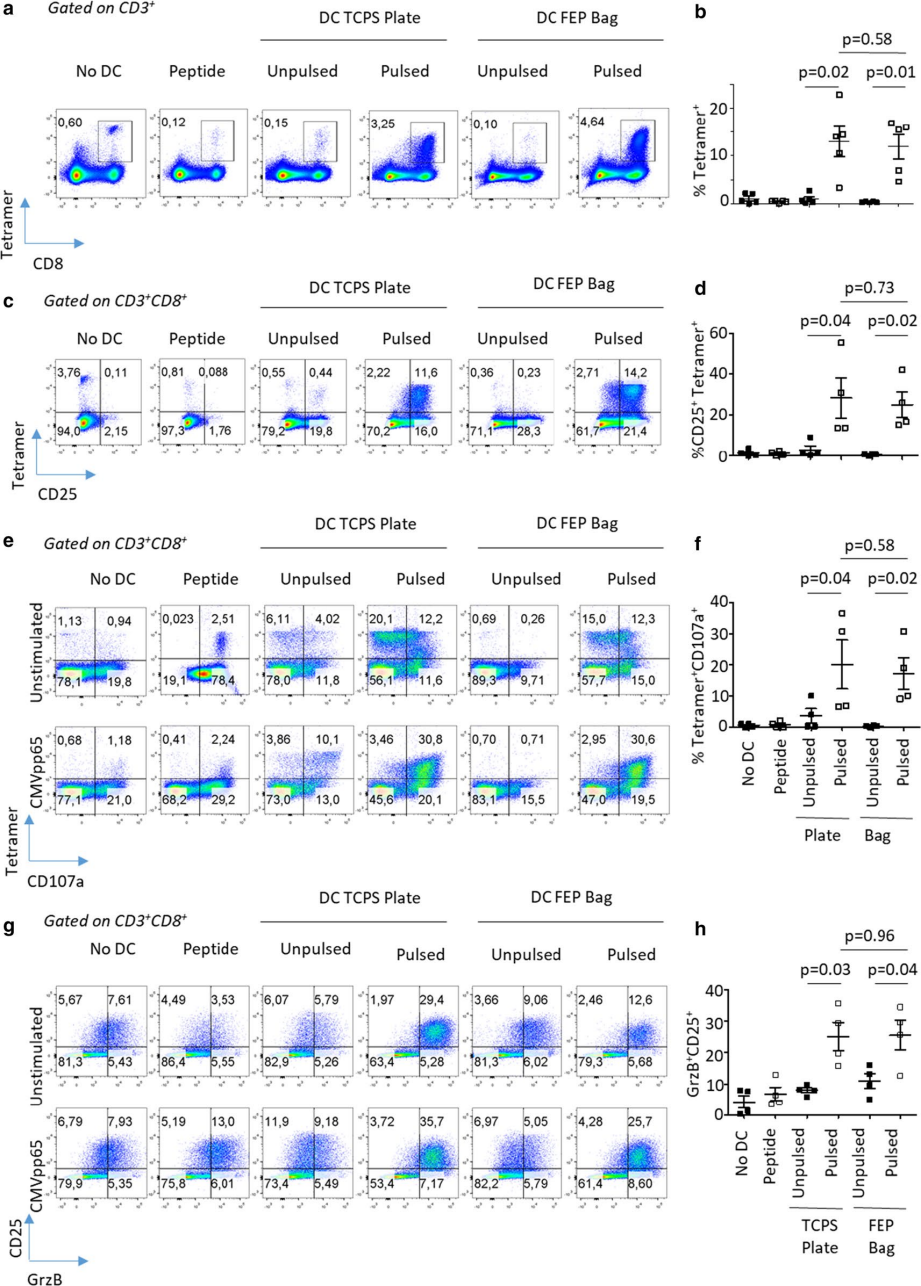
Pulsed Mo-DCs cultured in FEP bags or TCPS plates exhibit the same T cell activation capacity. Following Mo-DC differentiation, 15 × 10^6^ T cells were cultured in the presence of unpulsed or CMVpp65 peptide (NLV)-pulsed Mo-DC at 1:10 T cell: Mo-DC ratio. **a**, **b** At day 14, the proportion of NVL-specific T cells was assessed by flow cytometry. **c**, **d** The activation status of CD8^+^ T cells from the donor was assessed by the evaluation of CD25 expression. **e**, **f** The functionality of CD8^+^ T cells was evaluated through the expression of cytotoxic degranulation marker CD107a upon restimulation with CMVpp65 (NLV) antigen. **g**, **h** Granzyme B expression was evaluated upon re-stimulation with CMVpp65 (NLV) antigen. Panels show representative examples (**a**, **c**, **e**, **g**) or the mean ± SEM of 4 independent experiments (**b**, **d**, **f**, **h**). P-values shown are for paired comparisons

As adoptive T cell therapy requires infusion of a large number of T cells, the next question to address was whether Mo-DCs differentiated in TCPS plates or FEP bags could sustain and/or increase the frequency/number of antigen specific T cells beyond 7 days. To test this, we re-stimulated the day 7 T cell culture with freshly prepared Mo-DCs pulsed or not with NLV antigen. At day 14 of stimulation, the frequency of tetramer-specific CD8^+^ T cells was significantly higher in T cells cultured in the presence of NLV peptide pulsed Mo-DCs irrespective of the vessel used to generate the Mo-DCs (Fig. 6). In contrast, the frequency of tetramer-specific CD8^+^ T cells remained unchanged when T cells were exposed to unpulsed Mo-DCs or NLV peptide alone (Fig. 6a, b). Importantly, no statistically significant differences between TCPS plates or FEP bags were noted when quantifying the frequency of tetramer-specific CD8^+^ T cells (Fig. 6a, b). In both culture vessels, as observed after the first round of stimulation, CD25 expression was maintained on tetramer-specific CD8^+^ T cells cultured in presence of NLV-pulsed Mo-DCs whereas nonspecific T cells only displayed a modest increase in CD25 expression (Fig. 6c, d; Additional file 1: Figure S7). These results suggest that Mo-DCs differentiated in TCPS plates or FEP bags display similar capacity to activate and expand antigen-specific T cells.

To assess whether the activated tetramer specific CD8^+^ T cells were functionally competent, we studied their cytotoxic capacity by determining their antigen-induced degranulation status as well as granzyme B expression. First, following NLV peptide stimulation, we observed a significant upregulation, compared to unstimulated T cells, of cytotoxic degranulation marker CD107a in the tetramer-specific CD8^+^ T cells (Fig. 6e, f, Additional file 1: Figure S7C, F). Conversely, nonspecific CD4^+^ T cells and CD8^+^Tetramer^−^ T cells failed to degranulate upon restimulation with CMVpp65 peptide. These findings underline the specificity of this activation (Additional file 1: Figure S7A, B, D, E). In agreement with CD107a degranulation data, we observed a significant increase in granzyme B expression in CD25^+^CD8^+^ T cells activated in the presence of NLV peptide pulsed Mo-DCs (Fig. 6g, h). Importantly, the method of Mo-DC generation, whether produced in TCSP plates or FEP bags, did not significantly impact the cytotoxic potential of activated tetramer specific CD8^+^ T cells.

## Discussion

Harnessing the power of monocyte-derived DCs as a cell therapy product has shown substantial promise. Mo-DCs were approved in 2010 as the first prostate-cancer vaccine, sipuleucel-T, (Provenge^®^, Valeant Pharmaceuticals) by the FDA [25]. While this pioneer product showed modest success in overall survival benefit compared to placebo [26], its commercial success was limited by high production costs and logistic challenges [27]. Optimization of DC production methods is required to improve cell product consistency, quality and efficacy in order to benefit a broader patient population [28]. Renewed interest in DC-based cancer vaccines has been fuelled by the potential to combine this approach with other pharmacological or cell-based interventions aiming to promote the rejection of tumors [29].

Conventional methods for Mo-DC generation include a phase of TCPS plastic adherence for monocyte selection culture, followed by a maturation phase utilizing only the non-adherent cells, which are presumed to be ‘competent’ DCs [11, 30–32]. Since most clinical protocols of Mo-DC based immunotherapy require repeated injections from a large batch divided into smaller aliquots with identical properties, Mo-DC production through plastic requires large flask volumes which require large incubator space and time-consuming manual handling procedures. In addition to reducing the risks of contamination, closed culture vessels such as FEP bags reduce the volumes and handling steps required [33] as most cells remain in suspension [9].

To date, it is unknown whether the ‘ideal’ culture surface would promote monocyte adhesion or not [8]. In our hands, monocytes adhered both to FEP and TCPS surfaces and later detached from the surfaces during DC maturation. Cell detachment was coupled with cell aggregation, suggesting a transition from cell-material interactions to cell–cell binding, mirroring previous observations by Kurlander et al*.* who observed higher proportions of aggregated cells in FEP bags than TCPS cultures [9]. Interestingly, the reduced ability of CD14 + monocytes to adhere in FEP bags did not impact Mo-DC surface marker expression profiles, IL-10 or IL-12 expression or cell viability. Contrary to earlier findings with polyolefin bags [7, 22], the frequency of CD1a^+^ cells was not significantly different between Mo-DCs produced in TCPS plates or FEP bags (Fig. 3). Monocytes cultured both in FEP bags or TCPS plates differentiated into professional DCs as judged by their comparable capacity to generate functionally-competent antigen-specific cytotoxic CD8^+^ T cells. Our functional findings are consistent with our observations when studying protein adsorption on different model surfaces: the abundance of albumin in our medium led to the formation of a secondary protein layer with very similar mechanical properties and topography on polystyrene and fluoropolymer surfaces [34]. These studies were conducted in serum-free media and protein adlayer properties may differ between TCPS and FEP with media that contain serum (e.g. Kurlander et al*.* [9]) or in the absence of albumin [35].

In this study, HLA-02 restricted epitopes were used, indicating that CD8^+^ T cell activation was targeted. As most individuals have been exposed to virus such as CMV, it is likely that the expanded T cells emerged from the memory T cell pool. Further, Mo-DC have recently successfully been used to induce viral-specific T cell expansion in virus-naïve donors [36]. This technology could be valuable for a variety of applications such as cancer immunotherapy [2, 13], the expansion of tumor or viral reactive T cells, to treat, respectively, cancer or infection-prone immunocompromised patients [37, 38] as well as expanding other tolerogenic cells such as regulatory T cells (Tregs) in the context of auto-immunity or graft rejection [39].

Culture in bags usually involves high volumes that limit their applicability. In contrast, the research-scale closed culture systems and Mo-DC handling methods reported here can enable exploratory studies in many settings. Using the same closed culture bag systems for both R&D- and clinical-scale cell manufacturing and testing will also allow users to harmonize their culture and quality control protocols and thereby reduce the costly and time-consuming need for re-validation of all cell culture procedures and materials upon translation into the clinic.

## Conclusions

In summary, our results demonstrate that Mo-DCs can be generated in FEP culture bags and TCPS plates and show comparable phenotype and functional capacities, including cytokine secretion and T cell stimulation. To our knowledge, we are the first to clearly demonstrate the specific T cell activation potency of viral antigen-loaded Mo-DCs produced in FEP bags. This process can also be performed in FEP bags of less than 10 mL working volume to increase experimental throughput and accelerate clinical translation of promising research findings. This opens up new avenues for safe, robust and scalable manufacturing of cell-based products for immunotherapy. Thorough characterization of the cell population produced in closed culture vessels will help accelerate regulatory approval and speed to market, thereby reducing the burden of disease for patients worldwide.

## Supplementary information

**Additional file 1.** Supplementary Figures.

## Data Availability

All data generated or analysed during this study are included in this published article and its additional files.

## Abbreviations

FEP: Fluorinated ethylene propylene
Mo-DCs: Monocyte-derived dendritic cells
TCPS: Tissue culture polystyrene

## References

[1] U.S. Department of Health and Human Services FaDA. Sterile drug products produced by aseptic processing–current good manufacturing practice. In: Center for Drug Evaluation and Research (CDER) CfBEaRC, Office of Regulatory Affairs (ORA), editor. Rockville2004.

[2] Bastien JP, Minguy A, Dave V, Roy DC (2019). Cellular therapy approaches harnessing the power of the immune system for personalized cancer treatment. Semin Immunol.

[3] Sabado RL, Balan S, Bhardwaj N (2017). Dendritic cell-based immunotherapy. Cell Res.

[4] Cannon MJ, Block MS, Morehead LC, Knutson KL (2019). The evolving clinical landscape for dendritic cell vaccines and cancer immunotherapy. Immunotherapy.

[5] Chapuis F, Rosenzwajg M, Yagello M, Ekman M, Biberfeld P, Gluckman JC (1997). Differentiation of human dendritic cells from monocytes in vitro. Eur J Immunol.

[6] O'Neill DW, Bhardwaj N. Differentiation of peripheral blood monocytes into dendritic cells. Curr Protoc Immunol. 2005;Chapter 22:Unit 22F 4. 10.1002/0471142735.im22f04s67 18432951

[7] Elias M, van Zanten J, Hospers GA, Setroikromo A, de Jong MA, de Leij LF (2005). Closed system generation of dendritic cells from a single blood volume for clinical application in immunotherapy. J Clin Apher.

[8] Guyre CA, Fisher JL, Waugh MG, Wallace PK, Tretter CG, Ernstoff MS (2002). Advantages of hydrophobic culture bags over flasks for the generation of monocyte-derived dendritic cells for clinical applications. J Immunol Methods.

[9] Kurlander RJ, Tawab A, Fan Y, Carter CS, Read EJ (2006). A functional comparison of mature human dendritic cells prepared in fluorinated ethylene-propylene bags or polystyrene flasks. Transfusion.

[10] Garritsen HS, Macke L, Meyring W, Hannig H, Pagelow U, Wormann B (2010). Efficient generation of clinical-grade genetically modified dendritic cells for presentation of multiple tumor-associated proteins. Transfusion.

[11] Suen Y, Lee SM, Aono F, Hou S, Loudovaris M, Ofstein G (2001). Comparison of monocyte enrichment by immuno-magnetic depletion or adherence for the clinical-scale generation of DC. Cytotherapy.

[12] Wong EC, Lee SM, Hines K, Lee J, Carter CS, Kopp W (2002). Development of a closed-system process for clinical-scale generation of DCs: evaluation of two monocyte-enrichment methods and two culture containers. Cytotherapy.

[13] Fekete N, Beland AV, Campbell K, Clark SL, Hoesli CA (2018). Bags versus flasks: a comparison of cell culture systems for the production of dendritic cell-based immunotherapies. Transfusion..

[14] Yang C, Tibbitt MW, Basta L, Anseth KS (2014). Mechanical memory and dosing influence stem cell fate. Nat Mater.

[15] Bernard J, Ittelet D, Christoph A, Potron G, Adjizian JC, Kochman S (1998). Adherent-free generation of functional dendritic cells from purified blood monocytes in view of potential clinical use. Hematol Cell Ther.

[16] Cao H, Verge V, Baron C, Martinache C, Leon A, Scholl S (2000). In vitro generation of dendritic cells from human blood monocytes in experimental conditions compatible for in vivo cell therapy. J Hematother Stem Cell Res.

[17] Eyrich M, Schreiber SC, Rachor J, Krauss J, Pauwels F, Hain J (2014). Development and validation of a fully GMP-compliant production process of autologous, tumor-lysate-pulsed dendritic cells. Cytotherapy.

[18] Jarnjak-Jankovic S, Hammerstad H, Saeboe-Larssen S, Kvalheim G, Gaudernack G (2007). A full scale comparative study of methods for generation of functional dendritic cells for use as cancer vaccines. BMC cancer.

[19] Sorg RV, Ozcan Z, Brefort T, Fischer J, Ackermann R, Muller M (2003). Clinical-scale generation of dendritic cells in a closed system. J Immunother.

[20] Macke L, Garritsen HS, Meyring W, Hannig H, Pagelow U, Wormann B (2010). Evaluating maturation and genetic modification of human dendritic cells in a new polyolefin cell culture bag system. Transfusion.

[21] Tan YF, Sim GC, Habsah A, Leong CF, Cheong SK (2008). Experimental production of clinical-grade dendritic cell vaccine for acute myeloid leukemia. Malays J Pathol.

[22] Thurner B, Roder C, Dieckmann D, Heuer M, Kruse M, Glaser A (1999). Generation of large numbers of fully mature and stable dendritic cells from leukapheresis products for clinical application. J Immunol Methods.

[23] Laforte V, Lo PS, Li H, Juncker D (2017). Antibody colocalization microarray for cross-reactivity-free multiplexed protein analysis. Methods Mol Biol.

[24] Jonuleit H, Kuhn U, Muller G, Steinbrink K, Paragnik L, Schmitt E (1997). Pro-inflammatory cytokines and prostaglandins induce maturation of potent immunostimulatory dendritic cells under fetal calf serum-free conditions. Eur J Immunol.

[25] Anassi E, Ndefo UA (2011). Sipuleucel-T (provenge) injection: the first immunotherapy agent (vaccine) for hormone-refractory prostate cancer. Pharm Ther.

[26] George DJ, Nabhan C, DeVries T, Whitmore JB, Gomella LG (2015). Survival outcomes of sipuleucel-T phase III studies: impact of control-arm cross-over to salvage immunotherapy. Cancer Immunol Res.

[27] Ledford H (2015). Therapeutic cancer vaccine survives biotech bust. Nature.

[28] Galati D, Zanotta S (2018). Empowering dendritic cell cancer vaccination: the role of combinatorial strategies. Cytotherapy.

[29] Saxena M, Bhardwaj N (2018). Re-emergence of dendritic cell vaccines for cancer treatment. Trends Cancer.

[30] Tazbirkova A, Okai M, Horley DC, Crough TM, Maksoud A, Nieda M (2003). Effects of leukapheresis protocol, cell processing and cryopreservation on the generation of monocyte-derived DC for immune therapy. Cytotherapy.

[31] Pullarkat V, Lau R, Lee SM, Bender JG, Weber JS (2002). Large-scale monocyte enrichment coupled with a closed culture system for the generation of human dendritic cells. J Immunol Methods.

[32] Gulen D, Abe F, Maas S, Reed E, Cowan K, Pirruccello S (2008). Closing the manufacturing process of dendritic cell vaccines transduced with adenovirus vectors. Int Immunopharmacol.

[33] Mu LJ, Gaudernack G, Saeboe-Larssen S, Hammerstad H, Tierens A, Kvalheim G (2003). A protocol for generation of clinical grade mRNA-transfected monocyte-derived dendritic cells for cancer vaccines. Scand J Immunol.

[34] Wargenau A, Fekete N, Beland AV, Sabbatier G, Bowden OM, Boulanger MD (2019). Protein film formation on cell culture surfaces investigated by quartz crystal microbalance with dissipation monitoring and atomic force microscopy. Colloids Surf B Biointerfaces.

[35] Babaei S, Fekete N, Hoesli CA, Girard-Lauriault PL (2018). Adhesion of human monocytes to oxygen- and nitrogen- containing plasma polymers: effect of surface chemistry and protein adsorption. Colloids Surf B Biointerfaces.

[36] Hanajiri R, Sani GM, Hanley PJ, Silveira CG, Kallas EG, Keller MD (2019). Generation of Zika virus-specific T cells from seropositive and virus-naive donors for potential use as an autologous or "off-the-shelf" immunotherapeutic. Cytotherapy..

[37] Janelle V, Carli C, Taillefer J, Orio J, Delisle JS (2015). Defining novel parameters for the optimal priming and expansion of minor histocompatibility antigen-specific T cells in culture. J Transl Med.

[38] Granados DP, Rodenbrock A, Laverdure JP, Cote C, Caron-Lizotte O, Carli C (2016). Proteogenomic-based discovery of minor histocompatibility antigens with suitable features for immunotherapy of hematologic cancers. Leukemia.

[39] Veerapathran A, Pidala J, Beato F, Yu XZ, Anasetti C (2011). Ex vivo expansion of human Tregs specific for alloantigens presented directly or indirectly. Blood.

