# Supplementary material for: Closing the system: production of viral antigen-presenting dendritic cells eliciting specific CD8+ T cell activation in fluorinated ethylene propylene cell culture bags
Source: J Transl Med. 2020 Oct 9;18:383. doi: 10.1186/s12967-020-02543-1 (PMC7547414; doi:10.1186/s12967-020-02543-1)
Supplement: Supplementary file 1 — Additional file 1. Supplementary Figures. [file 12967_2020_2543_MOESM1_ESM.pdf]

Supplementary Figures

Closing the system: Production of Viral Antigen-Presenting Dendritic Cells Eliciting Specific $CD8^+$ T Cell Activation in Fluorinated Ethylene Propylene Cell Culture Bags

Jean-Philippe Bastien[2*], Natalie Fekete[1*], Ariane V. Beland[1], Marie-Paule Lachambre[2], Veronique Laforte[3-5], David Juncker[3-5], Vibhuti Dave[2,6], Denis-Claude Roy[2,7§], Corinne A. Hoesli[1,3§]

[1]Department of Chemical Engineering, McGill University, Montreal, Canada
[2]Division of Hematology-Oncology, Hopital Maisonneuve-Rosemont Research Center, Montreal Canada
[3]Department of Biomedical Engineering, McGill University, Montreal, Canada
[4]McGill Genome Centre, McGill University, Montreal, Canada
[5]Department of Neurology and Neurosurgery, McGill University, Montreal, Canada
[6]Department of Microbiology, Infectiology and Immunology, Université de Montréal, Montreal, Canada
[7]Department of Medicine, Université de Montréal, Montreal, Canada
*Equal contribution
[§]Co-senior authors

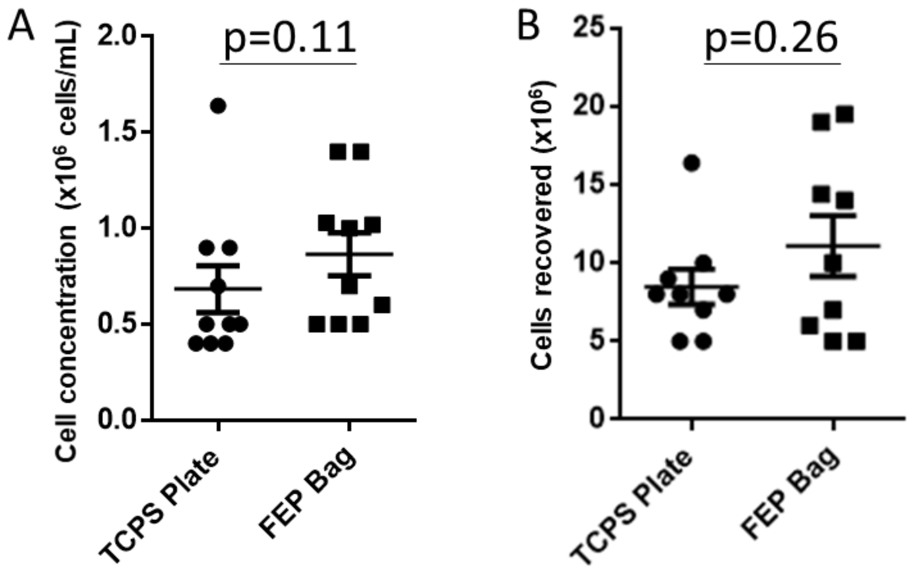

Figure S1. Yield of Mo-DCs after differentiation and maturation using NLV peptide. Final viable cell concentration (A) and final total viable cell number (B) of Mo-DCs after 7 days of culture starting from 30 x 10^6 monocytes initially seeded at 2 x 10^6 cells per mL into TCPS plates or FEP bags.

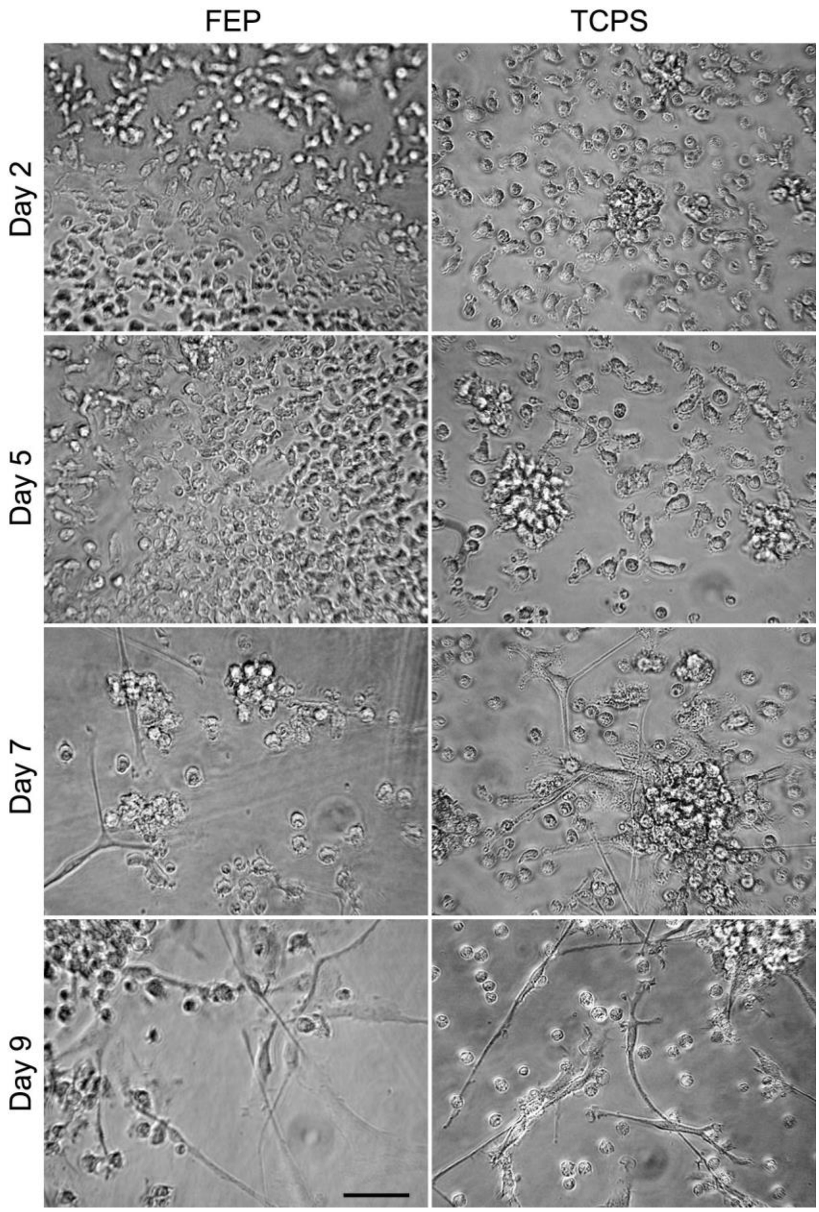

Figure S2. Morphological changes during the transition from monocyte (day 2) to immature Mo-DC (Day 5 and Day 7) and then mature DCs (Day 9) according to the maturation protocol using LPS treatment (**Erreur ! Source du renvoi introuvable.**A).

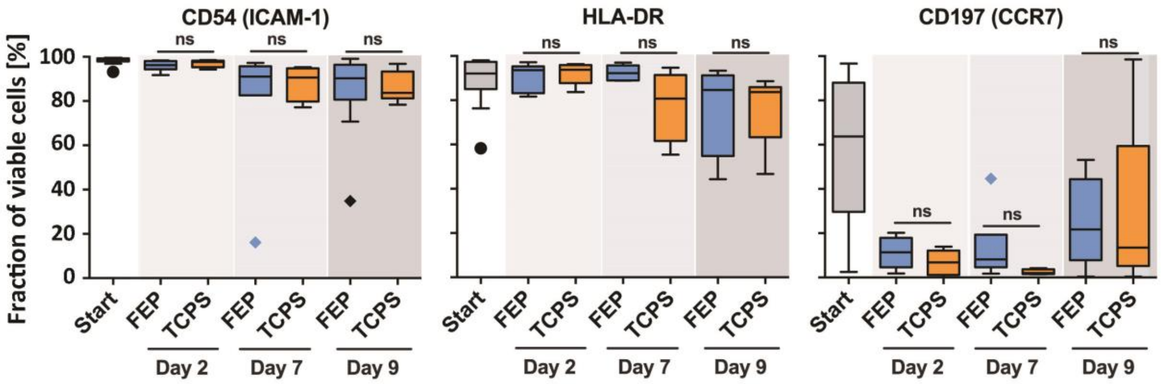

Figure S3. Mo-DCs can efficiently be generated on FEP bags and TCPS plates and show comparable surface marker expression. Flow cytometry was performed on monocytes before culture (Start) and after 2, 7 and 9 days of differentiation and maturation culture on FEP or TCPS surfaces. Shown are Tukey's Box-whiskers plots. ns: no statistically significant differences for both paired (n=4 donors for all surface markers at all time points except n=3 for CD86) and unpaired (n=13 donors at day 0; n = 4 to 10 donors at other time points) comparisons.

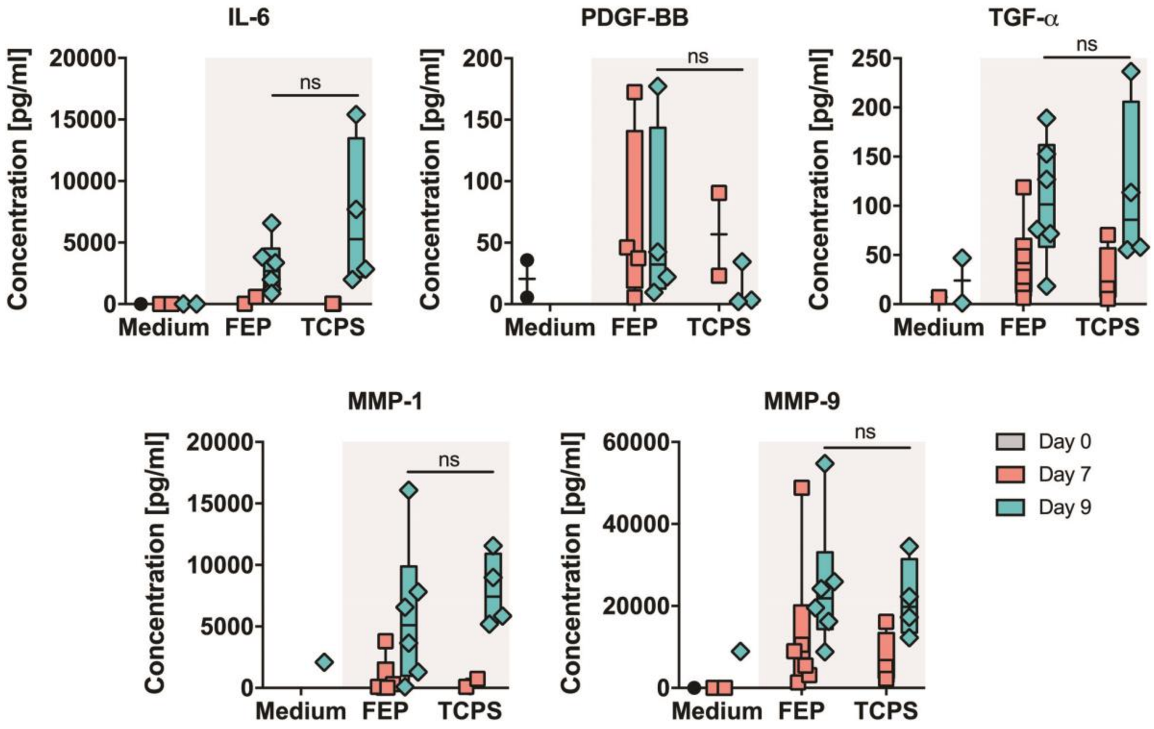

Figure S4. Mo-DCs produce signalling molecules and ECM-degrading proteins. Mo-DCs were cultured for 9 days in FEP culture bags or TCPS plates. Levels of IL-6, PDGF-BB, TGF-α, MMP-1 and MMP-9 in cell culture supernatants collected at day 7 and 9 of culture were compared to medium controls. Shown are min-max Box-and-whisker plots with individual data points (n = 6 for FEP; n = 4 for TCPS). ns: no statistically significant differences for both paired and unpaired comparisons.

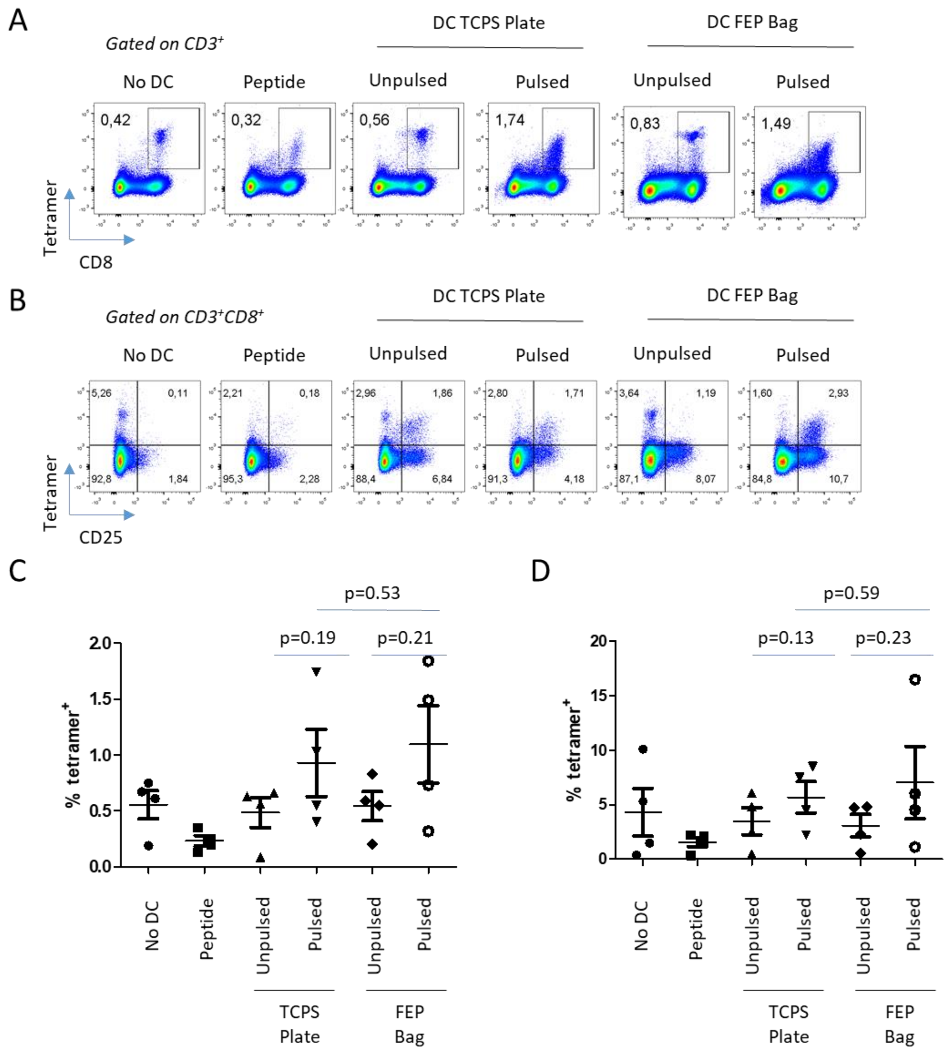

Figure S5. Pulsed Mo-DCs cultured in FEP bags or TCPS plates exhibit the same T cell activation capacity. Following Mo-DC differentiation, 15 x 10^6 T cells were cultured in the presence of unpulsed or CMVpp65 peptide (NLV)-pulsed Mo-DC at 1:10 T cell: Mo-DC ratio. (A, C) Proportion of NVL-specific T cells (CD3+) was assessed by flow cytometry at day 7. (B, D) Activation status of $CD8^+$ T cells through CD25 expression assessed by flow cytometry at day 7. Panels show representative examples (A and B) and the mean ± SEM of 4 independent experiments (C and D). P-values shown are for paired comparisons.

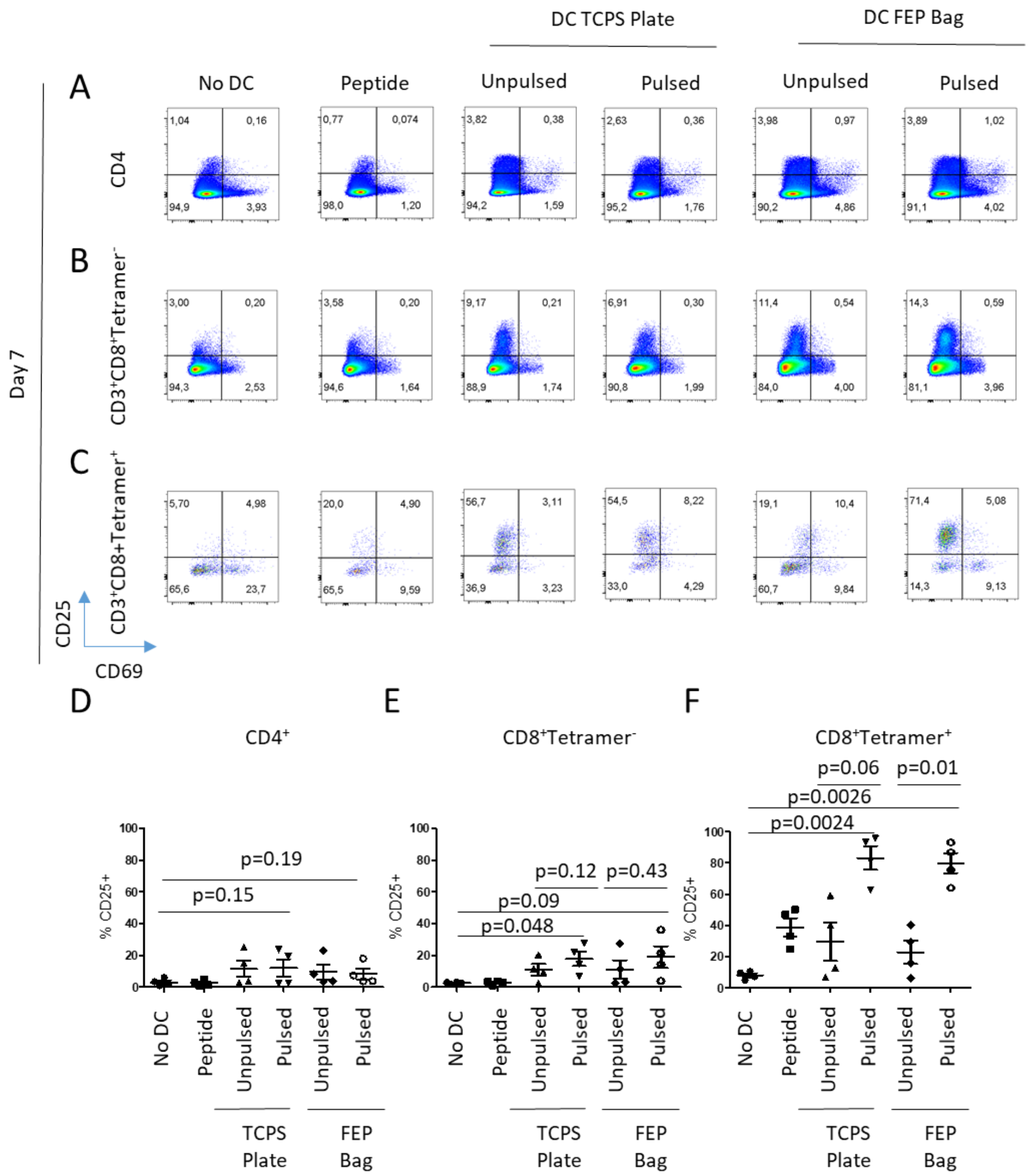

Figure S6. Pulsed Mo-DCs cultured in FEP bags or TCPS display similar antigen-specific and bystander T cell activation. Following Mo-DC differentiation, 15 x 10^6 T cells were cultured in the presence of unpulsed or CMVpp65 peptide (NLV)-pulsed Mo-DCs at 1:10 T cell: Mo-DC ratio. At day 7, the activation status assessed by the expression of CD25 and CD69 was evaluated on $CD4^+$ (A, D), $CD8^+Tetramer^-$ (B, E) and $CD8^+Tetramer^+$ (C, F) populations by flow cytometry. Panels show representative examples (A, B and C) and the mean ± SEM of 4 independent experiments (D, E and F). P-values shown are for paired comparisons.

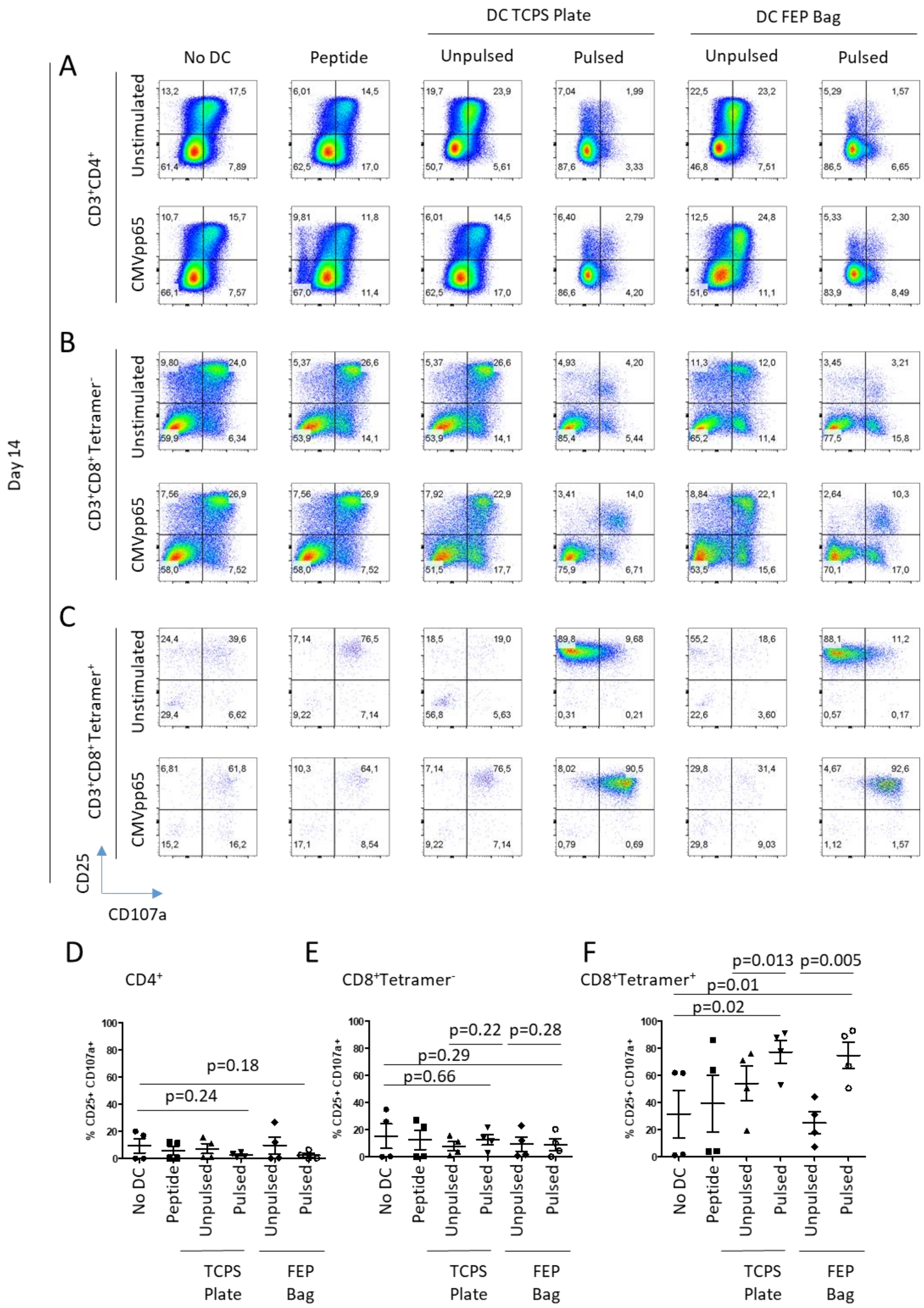

Figure S7. Tetramer specific cells undergo degranulation following restimulation with CMVpp65 peptide. Following Mo-DC differentiation, 15 x 10^6 T cells were cultured in the presence of unpulsed or CMVpp65 peptide (NLV)-pulsed Mo-DC at 1:10 T cell: Mo-DC ratio for 2 rounds of activation. At day 14, cells were stimulated with CMV pp65 peptide. The

activation status assessed by the expression of CD25 and functional status assessed by the expression of CD107a were evaluated on $CD4^+$ (A, D), $CD8^+Tetramer^-$ (B, E) and $CD8^+Tetramer^+$ (C, F) populations by flow cytometry. Panels show representative examples (A, B and C) and the mean ± SEM of 4 independent experiments (D, E and F). P-values shown are for paired comparisons.
